# stackPredAMR—a stacked random forest approach improves AMR phenotype prediction for multiple species and antimicrobial agents

**DOI:** 10.1093/bioadv/vbag153

**Published:** 2026-06-02

**Authors:** Julian Welling, Miriam Balzer, Leah Consten, Stefan Bletz, Jan Buer, Valerie Chapot, Dag Harmsen, Evelyn Heintschel von Heinegg, Alexander Mellmann, Wolfgang Pölking, Friederike Salhöfer, Frieder Schaumburg, Natalie Scherff, Niklas Wiesmann, Folker Meyer

**Affiliations:** Department of Medicine, University of Duisburg-Essen, Essen, 45147, Germany; Department of Computer Science, University of Duisburg-Essen, Essen, 45127, Germany; Department of Medicine, University of Duisburg-Essen, Essen, 45147, Germany; Department of Computer Science, University of Duisburg-Essen, Essen, 45127, Germany; Department of Medicine, University of Duisburg-Essen, Essen, 45147, Germany; Institute of Hygiene, University Hospital Münster, Münster, 48149, Germany; Department of Medicine, University of Duisburg-Essen, Essen, 45147, Germany; Department of Medicine, University of Duisburg-Essen, Essen, 45147, Germany; Ridom GmbH, Münster, 48149, Germany; Department of Medicine, University of Duisburg-Essen, Essen, 45147, Germany; Institute of Hygiene, University Hospital Münster, Münster, 48149, Germany; Institute of Hygiene, University Hospital Münster, Münster, 48149, Germany; Department of Medicine, University of Duisburg-Essen, Essen, 45147, Germany; Institute of Medical Microbiology, University Hospital Münster, Münster, 48149, Germany; Institute of Hygiene, University Hospital Münster, Münster, 48149, Germany; Institute of Medical Microbiology, University Hospital Münster, Münster, 48149, Germany; Department of Medicine, University of Duisburg-Essen, Essen, 45147, Germany; Department of Computer Science, University of Duisburg-Essen, Essen, 45127, Germany

## Abstract

**Motivation:**

Antimicrobial resistance is a growing global threat, creating a need for rapid and accurate antimicrobial susceptibility testing. Current phenotypic antimicrobial susceptibility testing methods rely on prior isolation and cultivation, making them time-consuming. Whole genome sequencing combined with machine learning offers a faster and cost-effective alternative, but existing approaches are often limited in species coverage, antimicrobial scope, or data availability.

**Results:**

We developed stackPredAMR, a machine learning framework for predicting resistance to 18 antimicrobial agents in three clinically important bacterial species: *Escherichia coli*, *Klebsiella pneumoniae*, and *Acinetobacter baumannii*. The model uses antimicrobial resistance gene presence as input and incorporates cross-resistance patterns through a stacked architecture with two random forest layers. Benchmarking on more than 2500 publicly available whole genome sequencing datasets with linked phenotypic resistance data showed strong performance, achieving a median accuracy of 0.94, ROC AUC of 0.97, and F1-score of 0.91, outperforming previously published methods. stackPredAMR is freely available and designed to support future extension to additional species and antimicrobial agents.

**Availability and implementation:**

Source code and datasets (database-driven reference approach, sample lists, and input features) are available at WIN-KID repository (https://github.com/IKIM-Essen/WIN-KID/tree/v1.0.0.0) and the release page (https://github.com/IKIM-Essen/WIN-KID/releases/tag/v1.0.0.0).

## 1 Introduction

Antimicrobial resistance (AMR) poses a growing threat to global health, with the highest burdens in low-resource settings (Murray *et al*. 2022). This threat is intensified by the relentless adaptability of microorganisms, a process accelerated by the widespread misuse and overuse of existing antimicrobial agents (Muteeb *et al*. 2023). Phenotypic antimicrobial susceptibility testing (AST) remains the gold standard for identifying resistance, requiring bacterial isolates and culturing in the presence of antimicrobial agents. This process typically takes from several days to several weeks for slow-growing species (Arnold *et al*. 2025). Whole genome-based AST (WAST) has the potential for faster prediction of resistance phenotypes, while simultaneously generating rich data for epidemiological surveillance (Su *et al*. 2019). To unlock the full potential of WAST, recent research increasingly focuses on machine learning (ML) approaches that can integrate complex genomic features and improve the accuracy and applicability of resistance prediction (Drouin *et al*. 2019, Bortolaia *et al*. 2020, Nguyen *et al*. 2020, Van Camp *et al*. 2020, Kim *et al*. 2022, Madden *et al*. 2024, Nsubuga *et al*. 2024, Zhou *et al*. 2024). While WGS (whole genome sequencing) still depends on cultured isolates, it is the first step toward culture-independent molecular diagnostics. Combined with direct metagenomic shotgun sequencing of patient samples and the computation of metagenome assembled genomes (Setubal 2021), it has the potential to become an alternative in routine diagnostics that bypasses bacterial culture and isolation.

Previous studies have demonstrated AMR phenotypes inference directly from WGS data (Arnold *et al*. 2025). Early approaches predominantly relied on rule-based methods, which use curated knowledge to interpret the presence or absence of known resistance genes (Gordon *et al*. 2014, The CRyPTIC Consortium and the 100000 Genomes Project 2018). These methods perform well in species with a small number of well-characterized AMR encoding loci. However, their scalability is limited: they require extensive manual curation and a deep understanding of species-specific resistance mechanisms, making them difficult to scale across the growing number of pathogens encountered in clinical settings (Anahtar *et al*. 2021).

In contrast, ML approaches have emerged as a powerful and scalable alternative that can learn predictive patterns directly from data, without requiring explicit modeling of the underlying resistance mechanisms. When trained on sufficiently large and diverse datasets, ML models have been shown to achieve high predictive accuracy for AST (Anahtar *et al*. 2021). However, AMR prediction remains challenging due to incomplete phenotype data and limited generalizability across species and resistance profiles.

Comparative evaluations suggest that random forests (RFs) and convolutional neural networks (CNNs) generally outperform other common classifiers, such as logistic regression or support vector machines, with AUCs up to 0.96 (Ren *et al*. 2022). Demonstrating the effectiveness of stacking in AMR prediction, AREScloud uses an ensemble of gradient boosting, logistic regression, and rule-based components trained on sequence features, and achieved 0.90 categorical agreement (Conzemius *et al*. 2022).

A major challenge in AMR phenotype prediction is incomplete phenotype data, as many samples lack antimicrobial susceptibility test results for multiple agents at different position in the dataset. Standard RF algorithms cannot natively handle such missing output values, which complicates the training of multi-agent models. Multi-label classification tools such as DeepAMR have shown that the cross-resistance pattern between agents significantly influences the prediction and should therefore not be omitted (Yang *et al*. 2019). While some RF studies, as such as that from Aytan-Aktug *et al*. (2020), have addressed this issue through phenotype imputation, this approach introduces uncertainty and relies on assumptions about the missing data. To avoid the need for imputation while still leveraging cross-resistance patterns, we developed stackPredAMR (stacked prediction of AMR), a stacked RF architecture. It leverages the well-established performance of RF models for AMR prediction and the demonstrated strengths of stacked approaches reported in prior work, combining them in a novel stacked model architecture that explicitly accommodates incomplete susceptibility data across multiple species and resistance profiles. In this framework, individual RF models are trained for each antimicrobial agent using available labels, and their predictions are then combined in a second-layer meta-model that captures shared resistance patterns across antimicrobial agents. In this way, we can model co-resistance structures more effectively, as the information from many antimicrobial agents and species flow into the same model, improving overall prediction performance.

## 2 Methods

### 2.1 Data collection and composition

We compiled a dataset of >3000 bacterial WGS samples from the BV-BRC (Olson *et al*. 2023). BV-BRC provides bacterial WGS data as assembled genomes and curated metadata such as AST profiles. Here, we included only samples for which both WGS data and AST information were available. For phenotypic information, only minimum inhibitory concentrations (MICs), i.e. the lowest concentration that inhibits bacterial growth, measured by the VITEK 2 system (bioMérieux, Marcy-l’Étoile, France) (Ling *et al*. 2001), were considered to ensure high quality and comparability of the data. Samples with antimicrobial agents that only occurred once were also excluded.

To ensure predictive performance, clinical relevance, and focus on high-priority pathogens, we restricted our performance evaluation to three bacterial species with sufficient data quantity: *Escherichia coli*, *Klebsiella pneumoniae*, and *Acinetobacter baumannii*. These species are included in the World Health Organization’s Bacterial Priority Pathogens List (World Health Organization 2024), highlighting their global importance due to their prevalence in healthcare-associated infections and widespread AMR. In contrast, Gram-positive pathogens were not included in this study due to limited and insufficiently balanced data for clinical relevant species in the available dataset, which would not have allowed for a comparable and statistically robust analysis across species.

Each sample in our dataset thus consists of paired WGS and AST, allowing training and evaluation of models that predict resistance phenotypes. AST data were collected for up to 18 antimicrobial agents across various drug classes, providing a broad and clinically meaningful basis for AMR prediction.

As proof of concept, we analyzed 20 additional samples that were sequenced and phenotyped locally, 10 each from the University Hospital Essen (Essen, Germany) (UME) and University Hospital Münster (Münster, Germany) (UKM). With 11 *Enterobacter cloacae* and 9 *Pseudomonas aeruginosa*, these samples consist of previously unstudied species to demonstrate the applicability of the approach to new species.

### 2.2 Data preprocessing

#### 2.2.1 Genomic data

Each WGS dataset was annotated for AMR encoding genes to derive genomic features, utilizing the Comprehensive Antibiotic Resistance Database (CARD) version 4.0.1. CARD is a curated repository that provides detailed molecular data on resistance genes, their corresponding resistance mechanisms, and associated phenotypic outcomes (Alcock *et al*. 2023). We aligned the assembled genome of each isolate against CARD’s set of validated AMR gene sequences to identify the presence of known AMR determinants using RGI 6.0.4 (https://card.mcmaster.ca/analyze/rgi). The properties Name, ORF ID, Antibiotic, DrugClass, and AMRGeneFamily from the database were then binary encoded for each gene. The resulting binary feature matrix contains the information for all samples, with each column representing a characteristic of an AMR gene and each row corresponding to a bacterial isolate (see Fig. 2 Preprocessed Data (1) CARD Results).

#### 2.2.2 Phenotypic data

Phenotypic AMR data were provided as MICs obtained through VITEK 2 testing. In addition, the organism type, which is routinely determined prior to antimicrobial susceptibility testing in clinical diagnostics, was available as part of the laboratory information. To standardize resistance categorization across antimicrobial agents and species, we interpreted MIC values according to the latest clinical breakpoints (v 15.0) published by EUCAST (European Committee on Antimicrobial Susceptibility Testing). The EUCAST thresholds used can be found in the Supplementary Materials, available as supplementary data at *Bioinformatics Advances* online (European Committee on Antimicrobial Susceptibility Testing (EUCAST) 2025). Each MIC was mapped to one of three clinical categories: Susceptible (S), Susceptible with increased exposure (I), or Resistant (R). The resulting panel of categorical susceptibility interpretations across all tested agents corresponds to the clinical antibiogram of an isolate. To filter out clear outliers, samples containing unique agent class combinations were filtered out. Agent-class combinations with <15 samples were excluded to ensure robust model training and evaluation. No further label balancing was performed in order to avoid reducing the already small sample size for some agents. The resulting dataset maintains a balanced distribution of samples across the three selected species and the 18 antimicrobial agents, allowing for meaningful inter- and intra-species comparisons in downstream analysis.

#### 2.3 Machine learning

We implemented our RF classification approaches using the scikit-learn Python library (Pedregosa *et al*. 2011). Initially, 20% of the samples were separated randomly to create a validation data set. The remaining dataset was divided into training and test sets using a stratified split based on the species, ensuring that the species distribution was preserved across both subsets. The separation of training and test data for 5-fold cross-validation was performed once at the beginning of each iteration and maintained throughout all modeling steps to avoid data leakage. The data set was not divided per species and all presented models are trained across three different species. The approach supports extending the data for additional species and antimicrobial agents. Furthermore, additional inputs can be easily added to train the models.

#### 2.3.1 CARD-driven reference approach

To demonstrate how effectively the CARD entries can predict resistance without further processing, a classification was made for all samples in the training and test datasets based directly on the annotation. Therefore, for each agent appearing in the “Antibiotics” column of the CARD results, an “R” was assigned, whilst all those not appearing were classified as “S”. To enable a comparison of these results with the VITEK results, all “I” samples were classified as “S”.

#### 2.3.2 One-layer classification approach

As a baseline, we implemented a standard one-layer RF model for AMR prediction. In this approach, a separate RF classifier was trained for each antimicrobial agent using its corresponding preprocessed input data. Since training was performed on unbalanced sets, each agent-specific model was only trained with the samples for which AST results were available. The training is identical to the “Training First Layer (3)” shown in Fig. 2, but the result with the highest probability is output directly. Each model predicts the antimicrobial susceptibility category (S/I/R) for a single antimicrobial agent based on the genotypic data and the organism type as features. The final output is a combined antibiogram, constructed by aggregating the individual predictions across all agent-specific models. This baseline serves as a reference to evaluate the added value of the stacked architecture.

#### 2.3.3 Stacked classification approach

In the first layer, a separate RF classifier was trained for each antimicrobial agent using samples for that antimicrobial agent. Each model took the preprocessed genomic data as input (i.e. binary encoded AMR gene characteristics) and returned the probability estimate for each resistance category as a multi-class output. The dataset is defined as:


(1) $$D^{\left(a\right)}=x_{i},y_{i}^{\left(a\right)}$$

with


(2) $$y_{i}^{\left(a\right)}\in \{S,I,R\}$$

with a∈A, where |*A*| = 18 corresponds to the number of considered antimicrobial agents and $y_{i}^{\left(a\right)}$ to the AST result for sample *i* of agent a. The feature vector $x_{i}$ for sample *i* is defined as:


(3) $$x_{i}=(x_{i\text{CARD}},x_{i\text{org}})\in R^{d}$$

for the first layer, where $x_{i\text{CARD}}$ denotes the binary encoded CARD features and $x_{i\text{org}}$ the numerically encoded organism identifier. Each model in the first layer is trained exclusively with samples for which AST results are available and the real-valued feature space allows no missing values in the feature vector of layer 1.

To robustly generate unbiased predictions for the next layer whilst avoiding data leakage, we applied an internal out-of-fold (OOF) procedure during the training of the first layer. These internal splits are part of the model training procedure and were not used for performance evaluation. This produced class probability estimates for each sample without including that sample in the training fold.

The output for each antibiotic *a* of the first layer is defined as:


(4) $$z_{i}^{\left(a\right)}=({\hat{P}}^{\left(a\right)}(S|x_{i}),{\hat{P}}^{\left(a\right)}(I|x_{i}),{\hat{P}}^{\left(a\right)}(R|x_{i}))\in R^{3}$$

with


(5) $$\;a\in A_{\text{AST}}$$

representing the predicted class probabilities for the three AST resistance categories (S/I/R) for sample *i* and antibiotic *a*. Let $A_{\text{AST}}\subseteq A$ denote the subset of antibiotics for which antimicrobial susceptibility testing (AST) results are available for the specific sample *i*.

The second layer aggregated the internal OOF predicted probabilities from all layer 1 models across antimicrobial agents into a new feature space. The feature vector $x_{i}$ for isolate *i* for the dataset of the second layer is therefore be defined as:


(6) $$x_{i}=(z_{i}^{\left(A\right)},x_{i\text{org}})$$

with


(7) $$z_{i}^{\left(A\right)}\in {\left(R\cup \{\text{NaN}\}\right)}^{d_{z}},\;x_{i\text{org}}\in R^{d_{\text{org}}}$$

where $z_{i}^{\left(A\right)}$ is defined as all probabilities vectors from layer 1 for all antibiotics *A* taken together, with missing predictions replaced by NaN (Not a Number). This allowed the model to capture potential relationships and co-resistance patterns across antimicrobial agents. For each antimicrobial agent, a new RF model was trained using the combined layer 1 outputs as input. The final prediction for each sample and antimicrobial agent was taken as the class *c* with the highest predicted probability from the respective layer 2 (*L*2) model:


(8) $${\hat{y}}_{i}^{\left(a\right)}=\arg\underset{c\in \{R,I,S\}}{\max}{\hat{P}}_{L2}^{\left(a\right)}(y=c|x_{i})$$

**Figure 1:**
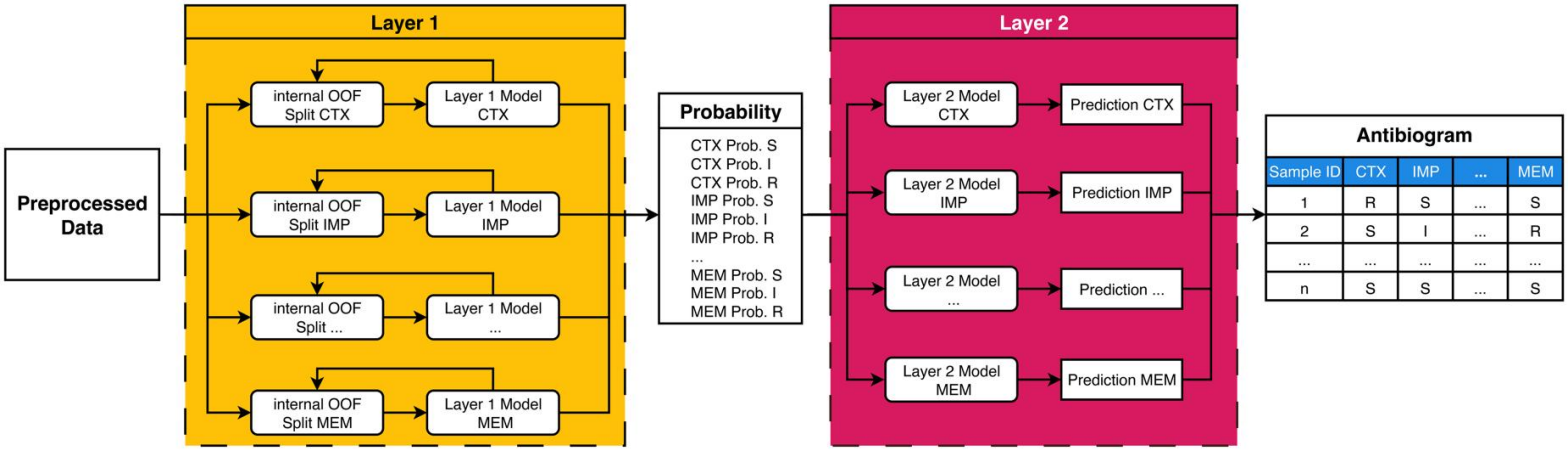
Stacked RF architecture overview, visualizing preprocessed data, first layer, second layer, and the final antibiogram. In the first layer for each antimicrobial agent (see Fig. 4 for the full forms of all abbreviations), a separate random forest classifier is trained on a split of the preprocessed data, generating internal out-of-fold (OOF) probability estimates for each resistance category (S/I/R). These probabilities are aggregated and used as input for a second layer, where for each antimicrobial agent new random forest models leverage cross-resistance patterns to produce a combined antibiogram.

The entire architecture is illustrated in Fig. 1.

**Figure 2:**
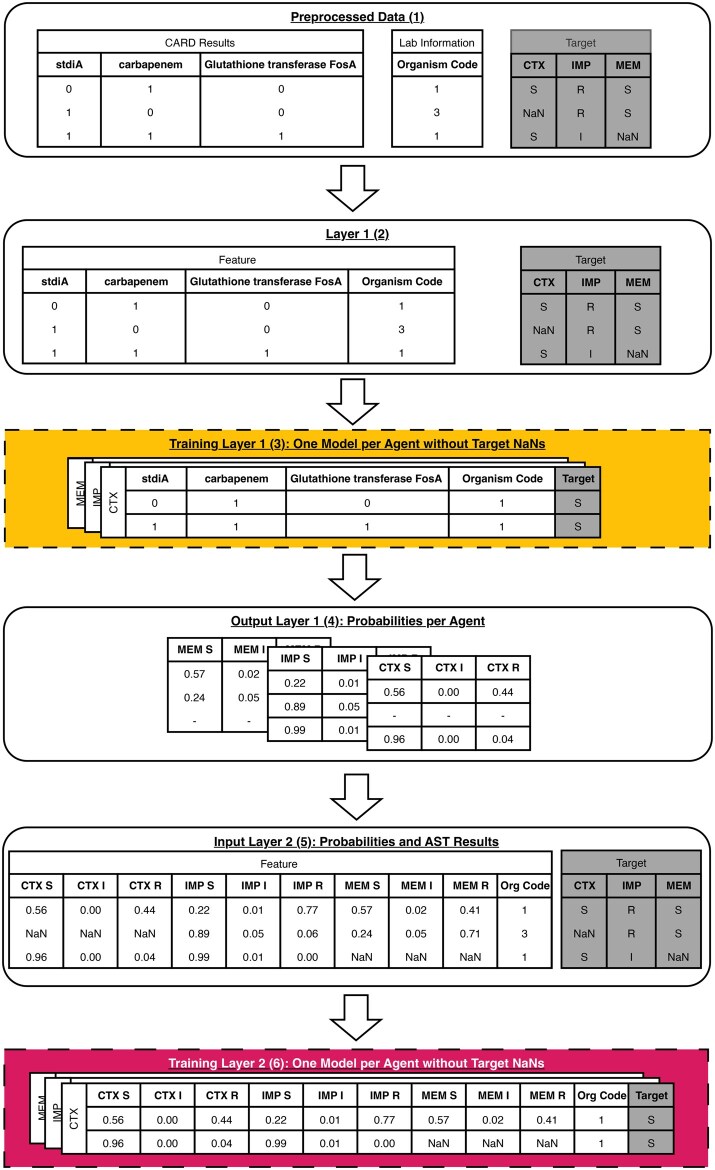
Stacked RF training overview, visualizing the training steps and handling of missing values in both layers. At the beginning, the Preprocessed Data (1), consisting of the binary encoded CARD results, laboratory information specifying the organism type in the organism code and the AST results for each antimicrobial agent (see Fig. 4 for the full forms of all abbreviations) as target are displayed. To be used as input for the first layer (2), the CARD results and lab information are combined as feature. For training of the first layer (3), feature and target are paired in a separate table for each agent, with target columns containing “NaN” being discarded. Each table is applied to train its agent’s specific model. The output of the first layer (4) consists of the probabilities for the different classes (S/I/R) of each agent. The probabilities of all agents paired with the organism code are combined as feature and used as input for the second layer (5). For all agent sample combinations for which no probability could be determined, “NaN” values are used, and the AST results from the first layer are applied again as target. For training of the second layer (6), feature and target are paired in a separate table for each agent, with feature columns retaining “NaN” values and target columns that contain “NaN” are discarded. Each agent-specific model is than trained with its specific table.

An important distinguishing feature is the handling of missing AST values during training. By using a separate RF model for each agent in the first layer, missing values for specific agents were removed without affecting the other models. The results of these models consisted of probabilities for the individual classes (S/I/R) of each agent and were summarized for each sample. These values were combined, with previously untreated values reported as NaN, and used as input for the second layer. Since within the second layer, each agent was trained on its proprietary model, missing target values were removed. In contrast, NaN values within the probabilities used as features for the RF models could be used for training and were retained. This approach enables the handling of missing AST values without losing the relationship between multiple AST results of one sample. The entire training process and handling of missing values is illustrated in Fig. 2.

#### 2.3.4 Model tuning and evaluation

To optimize model performance, we applied 5-fold cross-validation on the training set for hyperparameter tuning in both layers. Cross-validation was applied to the full model and is separate from the internal OOF procedure used in the first layer of the stacked model. The detailed RF settings used and a description of the hyperparameter tuning procedure can be found in the Supplementary Materials, available as supplementary data at *Bioinformatics Advances* online. Model evaluation was based on three commonly used classification metrics: accuracy, area under the receiver operating characteristic curve (ROC AUC), and F1-score. To determine ROC AUC and F1-score, the average value of all available classes for each antimicrobial agent was computed. Ensuring robust and representative performance estimates, we report the mean of these metrics across the held-out test sets of the 5-fold cross-validation from all folds, rather than relying on a single split. The resulting values were calculated for each species to check for possible differences between species. Singletons (antimicrobial agent classification combination occurring only once for a specific species) were excluded. This approach provides a more stable and reliable summary of the model’s generalization performance across different antimicrobial agents and bacterial species. In order to summarize the results of all antimicrobial agents while taking into account the different data set sizes for each agent, the sample-weighted median was calculated for each metric. Weighted bootstrapping was used to determine the 95% confidence interval (CI) for the weighted median. One-layer and stacked approach performances were compared using the Wilcoxon signed-rank test. Median differences and corresponding *P*-values were reported, with *P* ≤ 0.05 indicating statistically significant differences. The Python script for calculating weighted mean, CI, and Wilcoxon *P*-value can be found in the Supplementary Material, available as supplementary data at *Bioinformatics Advances* online. In order to enable comparison with as large a number of other publications, the unweighted median, hereinafter referred to as median, was also calculated. Feature importance scores from the second-layer RF classifiers were used to compute the contribution of individual features. In this context, each input feature corresponds to the predicted class probability and the species information from the first layer models. All probabilities of the classes of an antimicrobial agent were summed up in order to be able to determine which antimicrobial agents were used for the corresponding prediction. To predict results for the validation data set, the stacked approach was trained on the full cross-validation dataset 5 times and was evaluated with the same metrics. Additionally, very major error (VMEs; false susceptibility) and major error (ME; false resistance) were calculated. The mean value of all 5 validation runs was used for the results. Furthermore, the stacked approach was trained with all BV-BRC samples to predict the resistance of the 20 local sequenced samples from UME and UKM. For evaluation, their accuracy was calculated.

## 3 Results

### 3.1 Preprocessing results

By filtering samples with antimicrobial agents whose class occurred only once in the entire data set, two *Escherichia coli* samples were removed. Six agent-class combinations with <15 entries (Cefotaxime: I, Cefpodoxime: S, Fosfomycin: S/R, Moxifloxacin: S, Nitrofurantoin: S/R and Tigecycline S/R) were removed and in all cases except Cefotaxime, only one class remained, which led to the removal of the antimicrobial agent from the data set. After removing all samples for which AMR information was no longer available 2657 samples remained, of which 1315 were *Escherichia coli*, 928 *Klebsiella pneumoniae*, and 414 *Acinetobacter baumannii*.

The results of all three clinical classes (S/I/R) are available for 7 of the 18 antimicrobial agents investigated. For all others, the results are available for two classes. The distribution of the results across the classes is very divergent, with 44% S, 4% I, and 52% R over all species. The detailed results of the phenotype preprocessing are provided in Table 1.

**Table 1:** Results of the phenotype preprocessing of *Escherichia coli*, *Klebsiella pneumoniae*, and *Acinetobacter baumannii*.^a^

	S	I	R
**Amikacin**	1935	0	341
**Ampicillin**	76	0	1071
**Aztreonam**	189	43	539
**Cefazolin**	0	188	481
**Cefepime**	235	115	727
**Cefotaxime**	723	0	1041
**Ceftazidime**	1150	152	754
**Ceftriaxone**	173	0	697
**Cefuroxime**	0	159	544
**Ciprofloxacin**	810	101	1582
**Ertapenem**	424	0	621
**Gentamicin**	1649	0	882
**Imipenem**	241	23	644
**Levofloxacin**	71	175	736
**Meropenem**	1782	71	660
**Norfloxacin**	138	0	27
**Tobramycin**	692	0	625
**Trimethoprim**	67	0	164
**Sum**	10 355	1027	12 136
**Share**	0.44	0.04	0.52

a The table displays the number of samples in each category (S/I/R) for each antimicrobial agent. Counts are based on all available phenotype measurements per genome–drug combination (i.e. multiple entries per genome are possible). Sum refers to the total number of phenotype entries aggregated across all antimicrobial agents, and share denotes the relative proportion of each category across these total entries.

2125 randomly selected samples of the 2657 samples were used for cross-validation and 532 were used as the hold out validation data set.

Genotypic preprocessing resulted in 1257 binary encoded features.

### 3.2 Performance of CARD-driven reference approach

The results, which were derived directly from the CARD annotations without the use of machine learning, showed a median accuracy of 0.577 across all 18 agents. The median ROC AUC and F1 score were 0.696 and 0.503. A more detailed decomposition of these results can be found in the Supplementary Material, available as supplementary data at *Bioinformatics Advances* online.

### 3.3 Performance of one-layer RF Prediction

**Figure 3:**
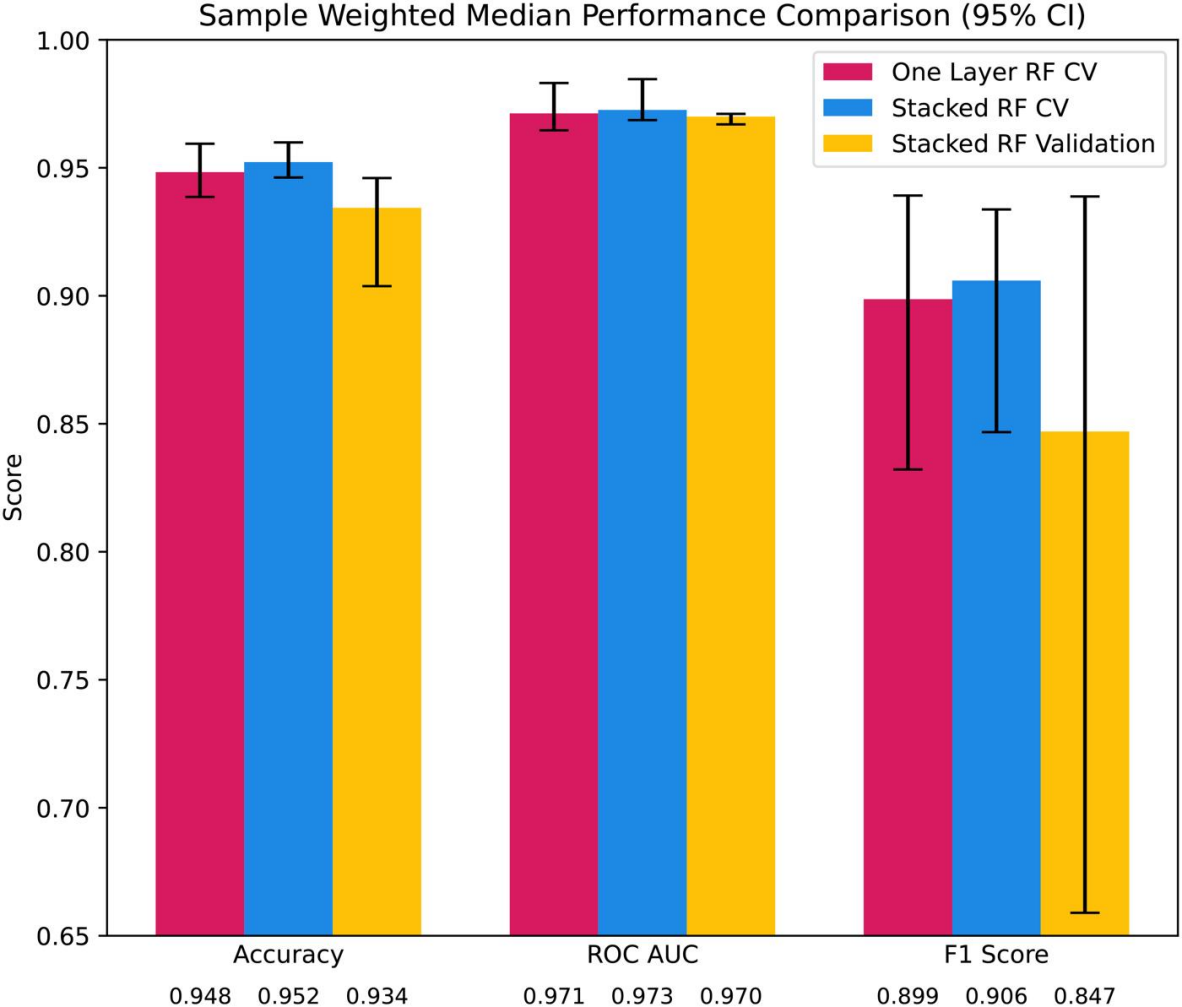
Sample weighted median performance comparison of one-layer RF CV, stacked RF CV and stacked RF validation (left to right). Accuracy, ROC AUC, and F1-score are displayed with the corresponding 95% CI as error bars. The detailed CI values can be found in the Supplementary Material, available as supplementary data at *Bioinformatics Advances* online.

The one-layer RF model was evaluated via cross-validation across 18 antimicrobial agents using accuracy, ROC AUC, and F1-score as performance metrics. The median accuracy was 0.949, the median ROC AUC was 0.970, and the median F1-score was 0.905. The sample-weighted median values with a 95% CI can be found in Fig. 3 with an accuracy of 0.948, ROC AUC of 0.971, and F1-score of 0.899. Performance varied across individual antimicrobial agents: The lowest accuracy was observed for Imipenem (0.877), while the highest was achieved for Cefotaxime (0.982). ROC AUC values ranged from 0.921 for Norfloxacin to 0.990 for Ceftriaxone, and F1-scores ranged from 0.699 for Meropenem to 0.981 for Cefotaxime. Detailed results for each antimicrobial agent are provided in Table 2.

**Table 2:** Cross-validation evaluation metrics of the one-layer RF classification across all antimicrobial agents.^a^

Antibiotic	Accuracy	ROC AUC	F1 score
**Amikacin**	0.948	0.980	0.899
**Ampicillin**	0.974	0.965	0.891
**Aztreonam**	0.954	0.971	0.935
**Cefazolin**	0.961	0.980	0.953
**Cefepime**	0.892	0.940	0.803
**Cefotaxime**	0.982	0.995	0.981
**Ceftazidime**	0.950	0.983	0.911
**Ceftriaxone**	0.980	0.990	0.968
**Cefuroxime**	0.969	0.974	0.957
**Ciprofloxacin**	0.939	0.969	0.832
**Ertapenem**	0.883	0.938	0.878
**Gentamicin**	0.959	0.987	0.956
**Imipenem**	0.877	0.947	0.740
**Levofloxacin**	0.922	0.966	0.866
**Meropenem**	0.900	0.946	0.699
**Norfloxacin**	0.961	0.921	0.924
**Tobramycin**	0.939	0.976	0.939
**Trimethoprim**	0.865	0.932	0.843
**Median**	0.949	0.970	0.905
**WT Median**	0.948	0.971	0.899

a The overall median is displayed before the last row. The last row shows the weighted median, which was weighted based on the total number of test samples for each individual agent. Cells are tint-coded according to performance thresholds: dark (>0.95), light (>0.85–0.95), and white (≤ 0.85).

### 3.4 Performance of stacked RF prediction

#### 3.4.1 Results per antimicrobial agent

We evaluated the stacked RF model with the same metrics as the one-layer baseline. The median accuracy across all antimicrobial agents was 0.953, the median ROC AUC reached 0.972, and the median F1-score was 0.925. The sample-weighted median values with a 95 % CI can be found in Fig. 3 with an accuracy of 0.952, ROC AUC of 0.973, and F1-score of 0.906. Performance across individual antimicrobial agents showed limited variability. The lowest accuracy was observed for Cefepime (0.884), while the highest was achieved for Cefotaxime (0.987). ROC AUC values ranged from 0.928 for Trimethoprim to 0.992 for Cefotaxime. F1-scores ranged from 0.737 for Imipenem to 0.987 for Cefotaxime. A complete overview of individual antimicrobial agent results can be found in Table 3.

**Table 3:** Cross-validation evaluation metrics of the stacked RF classification across all antimicrobial agents.^a^

Antibiotic	Accuracy	ROC AUC	F1 score
**Amikacin**	0.953	0.984	0.906
**Ampicillin**	0.976	0.972	0.902
**Aztreonam**	0.949	0.970	0.924
**Cefazolin**	0.976	0.984	0.970
**Cefepime**	0.884	0.963	0.798
**Cefotaxime**	0.987	0.998	0.987
**Ceftazidime**	0.957	0.987	0.925
**Ceftriaxone**	0.981	0.992	0.970
**Cefuroxime**	0.979	0.984	0.970
**Ciprofloxacin**	0.952	0.973	0.847
**Ertapenem**	0.934	0.976	0.931
**Gentamicin**	0.960	0.985	0.956
**Imipenem**	0.894	0.965	0.737
**Levofloxacin**	0.927	0.967	0.876
**Meropenem**	0.946	0.963	0.739
**Norfloxacin**	0.953	0.960	0.936
**Tobramycin**	0.934	0.969	0.934
**Trimethoprim**	0.905	0.928	0.890
**Median**	0.953	0.972	0.925
**WT Median**	0.952	0.973	0.906

a The overall median is displayed before the last row. The last row shows the weighted median, which was weighted based on the total number of test samples for each individual agent. Cells are tint-coded according to performance thresholds: dark (>0.95), light (>0.85–0.95), and white (≤ 0.85).

Table 4 shows the changes between the one-layer model and the stacked model. Changes ≥ 0.01 are highlighted by a tint. This shows that only the F1-score of Aztreonam has deteriorated considerably. In contrast, the F1-score shows a clear improvement for 11 agents. In terms of accuracy and ROC AUC, the values for 6 agents each improve and none deteriorate considerably. The biggest gain in accuracy and F1-score was made for Ertapenem with 0.051 and 0.053. Norfloxacin showed the biggest ROC AUC improvement with 0.039. Furthermore, the Wilcoxon signed-rank test revealed statistically significant differences between the two models across all evaluation metrics, with *P*-values of 0.030 for accuracy, 0.009 for ROC AUC, and 0.005 for the F1-score (all *P* ≤ 0.05).

**Table 4:** Difference between the evaluation metrics of the one-layer RF (Table 2) and the stacked RF (Table 3).^a^

	Accuracy	ROC AUC	F1 score
**Amikacin**	0.005	0.004	0.007
**Ampicillin**	0.002	0.007	0.011
**Aztreonam**	−0.005	−0.001	−0.011
**Cefazolin**	0.015	0.005	0.017
**Cefepime**	−0.008	0.023	−0.005
**Cefotaxime**	0.005	0.003	0.005
**Ceftazidime**	0.007	0.004	0.014
**Ceftriaxone**	0.001	0.002	0.002
**Cefuroxime**	0.009	0.010	0.013
**Ciprofloxacin**	0.014	0.003	0.015
**Ertapenem**	0.051	0.038	0.053
**Gentamicin**	0.001	−0.002	0.001
**Imipenem**	0.017	0.018	−0.003
**Levofloxacin**	0.005	0.001	0.010
**Meropenem**	0.046	0.016	0.040
**Norfloxacin**	−0.008	0.039	0.012
**Tobramycin**	−0.005	−0.007	−0.005
**Trimethoprim**	0.041	−0.005	0.047
**Median**	0.003	0.002	0.020
**WT Median**	0.004	0.001	0.007
**Wilcoxon *P*-value**	0.030	0.009	0.005

a A performance difference by ≥ 0.01 is indicated by a tint. The last row reports the Wilcoxon test *P*-value aggregation across all antimicrobial agents, indicating whether the performance differences between the two models are statistically significant. A *P*-value of ≤ 0.05 is considered significantly different.

#### 3.4.2 Feature importance

**Figure 4:**
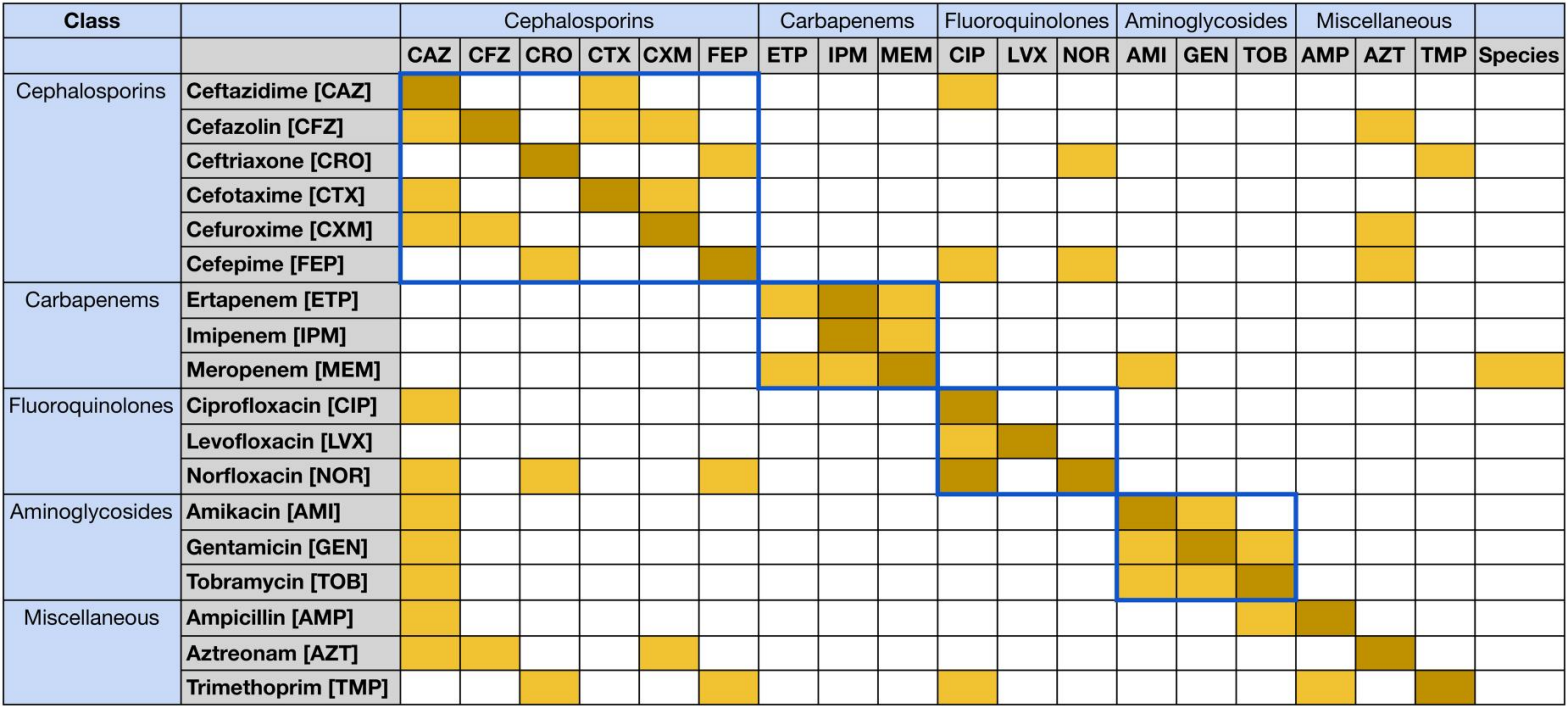
Mean feature importance of the second-layer CV of the stacked RF model. Rows list the antimicrobial agents for which the models were trained, with their full names followed by three-letter abbreviations. Columns represent the features, consisting of the set of all antimicrobial agents displayed by their abbreviations and the information of the bacterial species. Antimicrobial agents in the rows and columns are additionally assigned to their classes, with classes that only occur once being collected in “Miscellaneous.” Cells belonging to the same drug class are highlighted with a frame. Each cell indicates the contribution of the corresponding feature to the prediction of the given antimicrobial agent model. Intensity of the tint increases with the feature importance.: dark (≥ 0.25), light (≥ 0.05–0.25), and white (< 0.05). The exact values for each cell can be found in the Supplementary Material, available as supplementary data at *Bioinformatics Advances* online.

To gain insight into how the second layer of the stacked model integrates predictions from the first layer, we examined the mean feature importance scores of the stacked RF CV runs for each antimicrobial agent-specific classifier. These scores indicate the relative contribution of each input feature (i.e. class probabilities from the first layer models) to the final prediction. Detailed results of the feature importance analysis are provided in Fig. 4. The exact feature importance values can be found in the Supplementary Material, available as supplementary data at *Bioinformatics Advances* online.

For all antimicrobial agents, the first layer predicted probabilities for the same antimicrobial agent consistently ranked as the most important features in the corresponding second-layer model, except for Ertapenem where Imipenem ranked higher, indicated by the dark diagonal in Fig. 4. It is also noticeable that Ertapenem has the lowest feature importance for itself, with only 0.18, and Gentamicin has the highest at 0.68 for itself. The species feature was considered for 4 antimicrobial agents and achieved scores ranging from 0.01 to 0.07. Considering the values within the classes, 10 out of 30 Cephalosporins show a score of ≥ 0.05 among each other (see Fig. 4). For Fluoroquinolones, this value is exceeded in 2 out of 6 cases, and for Aminoglycosides and Cephalosporins in 5 out of 6 cases, respectively.

#### 3.4.3 Results per species

To evaluate model performance across different bacterial species, we analyzed the results separately for *Escherichia coli*, *Klebsiella pneumoniae*, and *Acinetobacter baumannii*. As shown in Table 5, the median accuracy was highest for *Escherichia coli* (0.956), followed by *Klebsiella pneumoniae* (0.942) and *Acinetobacter baumannii* (0.870). The median ROC AUC values were 0.976, 0.976, and 0.863 for *Escherichia coli*, *Klebsiella pneumoniae*, and *Acinetobacter baumannii*, respectively. In the median F1-score, *Klebsiella pneumoniae* performs best with 0.899, followed by *Escherichia coli* with 0.849 and *Acinetobacter baumannii* with 0.705. A more detailed decomposition of these results can be found in the Supplementary Material, available as supplementary data at *Bioinformatics Advances* online.

**Table 5:** Median evaluation metrics per species of the stacked RF model.

Species	Accuracy	ROC mean	F1 mean
***E. coli***	0.956	0.976	0.849
***K. pneumoniae***	0.942	0.976	0.899
***A. baumannii***	0.870	0.863	0.705

#### 3.4.4 Performance of validation dataset prediction

The stacked RF model generated predictions across 18 antimicrobial agents for the validation dataset, which were evaluated using accuracy, ROC AUC, and F1-score as performance metrics. The median accuracy was 0.938, the median ROC AUC was 0.971, and the median F1-score was 0.906. The sample-weighted median values with a 95 % CI can be found in Fig. 3 with an accuracy of 0.934, ROC AUC of 0.970, and F1-score of 0.847. The CI of the F1-score is notably large with 0.659–0.939. Performance varied across individual antimicrobial agents: The lowest accuracy was observed for Cefotaxime (0.585), while the highest was achieved for Cefuroxime (0.986). ROC AUC values ranged from 0.890 for Ertapenem to 0.993 for Ceftriaxone, and F1-scores ranged from 0.369 for Cefotaxime to 0.977 for Cefuroxime. Detailed results for each antimicrobial agent are provided in Table 6. ME and VME showed significant differences with medians of 1.19 and 3.77. Only three antimicrobial agents had a VME of >3 and 11 antimicrobial agents had an ME of >3. The detailed values can be found in the Supplementary Material, available as supplementary data at *Bioinformatics Advances* online.

**Table 6:** Evaluation metrics of the validation stacked RF classification across all antimicrobial agents.^a^

	Accuracy	ROC AUC	F1 score
**Amikacin**	0.941	0.970	0.934
**Ampicillin**	0.955	0.961	0.689
**Aztreonam**	0.963	0.966	0.911
**Cefazolin**	0.972	0.990	0.961
**Cefepime**	0.904	0.928	0.827
**Cefotaxime**	0.585	0.992	0.369
**Ceftazidime**	0.966	0.983	0.939
**Ceftriaxone**	0.976	0.993	0.966
**Cefuroxime**	0.986	0.970	0.977
**Ciprofloxacin**	0.925	0.967	0.627
**Ertapenem**	0.738	0.890	0.659
**Gentamicin**	0.946	0.971	0.940
**Imipenem**	0.876	0.900	0.556
**Levofloxacin**	0.934	0.971	0.847
**Meropenem**	0.904	0.971	0.709
**Norfloxacin**	0.973	0.978	0.962
**Tobramycin**	0.934	0.958	0.934
**Trimethoprim**	0.878	0.948	0.837
**Median**	0.938	0.971	0.906
**WT Median**	0.934	0.970	0.847

a The overall median is displayed before the last row. The last row shows the weighted median, which was weighted based on the total number of test samples for each individual agent. Cells are tint-coded according to performance thresholds: dark (>0.95), light (>0.85–0.95), and white (≤ 0.85).

The training of the stacked model for the validation dataset completed in 86.6 seconds and the prediction required 11.9 seconds. Detailed runtime values can be found in the Supplementary Material, available as supplementary data at *Bioinformatics Advances* online.

#### 3.4.5 Locally sequenced and phenotyped samples

The exemplary analysis of the 20 local samples (11 *E. cloacae* and 9 *P. aeruginosa*) showed large differences in the predictive performance of the individual antimicrobial agents for species not in our training set. AMR results from both sources were available for 10 antimicrobial agents, resulting in a weighted overall accuracy of 0.342. However, for the antimicrobial agents Amikacin, Tobramycin, and Gentamicin, good results were achieved with a combined weighted accuracy of >0.9 for both the UME and the UKM data, even without training the models for these species. In contrast, Cefotaxime, Ciprofloxacin, and Imipenem show poor results for both sources with a combined weighted accuracy of <0.4. The results for the remaining three antimicrobial agents are between 0.4 and 0.9. Detailed results are provided in Table 7.

**Table 7:** Accuracy of the locally sequenced and phenotyped samples from UME (University Hospital Essen) and UKM (University Hospital Münster).^a^

	Sample num.	Accuracy	Sample num.	Accuracy	Combined accuracy
	UKM		UME		
**Amikacin**	5	1.000	4	1.000	1.000
**Ampicillin**	5	1.000	–	–	–
**Aztreonam**	5	0.800	1	0.000	0.667
**Cefepime**	5	0.000	–	–	–
**Cefotaxime**	5	0.800	6	0.000	0.364
**Ceftazidime**	10	0.300	10	0.600	0.450
**Ciprofloxacin**	10	0.500	10	0.000	0.250
**Ertapenem**	5	0.600	–	–	–
**Gentamicin**	5	0.800	6	1.000	0.909
**Imipenem**	10	0.500	10	0.000	0.250
**Levofloxacin**	–	–	3	0.000	–
**Meropenem**	10	0.700	10	0.900	0.800
**Tobramycin**	5	1.000	4	1.000	1.000
**Trimethoprim**	5	0.400	–	–	–
**WT Median**	85	0.600	64	0.000	0.342

a The columns show the number of samples and the accuracy separately for UKM (left), UME (middle), and their combined accuracy (right). The antimicrobial agents for which predictions were made and their weighted median are shown in the rows.

## 4 Discussion

In this study, we developed and evaluated stackPredAMR, a stacked RF approach for predicting AMR phenotypes from bacterial WGS data. Using a dataset of over 2500 publicly available genomes for three high-priority pathogens (*Escherichia coli*, *Klebsiella pneumoniae*, and *Acinetobacter baumannii*), we compared a standard one-layer RF model to our stacked architecture. The stacked model integrates predictions from individual antimicrobial agent-specific models in a second-layer classifier, allowing it to capture cross-resistance patterns without requiring imputation of missing phenotypes. Performance was assessed using accuracy, ROC AUC, and F1-score across 18 antimicrobial agents and three species, with results reported per antimicrobial agent across all species. Additionally, the overall results were calculated for each individual species.

### 4.1 CARD-driven reference approach

Direct classification based on CARD results, with a median accuracy of just 0.577, performs only marginally better than chance. Although the complexity was reduced by removing the “I” class from this classification, the results clearly show that the CARD results alone, without further analysis, are not suitable for predicting resistance.

### 4.2 One-layer model

The one-layer RF model already demonstrated strong predictive performance, achieving a median accuracy of 0.949, median ROC AUC of 0.970, and a median F1-score of 0.905. The weighted medians deviate only slightly from these values, which suggests that the observed performance is robust to differences in sample size across antimicrobial agents and is not driven by a small subset of frequently tested agents. Notably, the F1-score exhibits a comparatively wide confidence interval, likely reflecting the substantial differences in the class distributions of the individual antimicrobial agents. Despite the input depending only on AMR gene presence, the results improve compared to those of more complex methods such as the k-mer approach of Jaillard *et al*. (2020) with a median AUC of 0.92. In contrast to the model presented here, the k-mer method is able to capture gene regulation, which appears to play only a secondary role in the prediction for the antimicrobial agents and species combinations investigated. Compared to the CARD-driven reference approach, there is a significant improvement, highlighting the benefits of analyzing this data using machine learning.

These results indicate that the presence or absence of known AMR genes—annotated using the CARD database—is highly informative for predicting the antimicrobial agent resistance phenotype. As all models were trained on all features derived from CARD annotations, the consistently strong performance suggests that they were able to learn meaningful associations between these genomic features and the observed resistance phenotypes. The strong performance across antimicrobial agents suggests that CARD provides comprehensive coverage for the resistance mechanisms relevant to the antimicrobial agents and species investigated. The slightly lower F1-scores compared to ROC AUC and accuracy may be attributed to class imbalance, both in terms of species representation and the distribution of resistance phenotypes (S/I/R) for certain antimicrobial agents.

### 4.3 Stacked model

The stacked RF model further improved overall performance, reaching a median accuracy of 0.953, median ROC AUC of 0.972, and median F1-score of 0.925. The weighted medians of accuracy and ROC AUC deviate only slightly from these values, but the weighted F1-score differs more considerably, with 0.906. As with the one-layer model, the confidence interval for this value is larger than for the other metrics. This indicates heterogeneous and threshold-sensitive minority-class performance among frequently tested antimicrobial agents, while overall discriminatory performance remains strong. Notably, even the lowest accuracy among all antimicrobial agents was 0.884, and the lowest ROC AUC was 0.928, suggesting robust predictive capability across all targets. A direct comparison with the one-layer model revealed statistically significant improvements across all three evaluation metrics when aggregated over all antimicrobial agents. While the stacked approach may yield slightly lower performance for individual cases, these results indicate superior overall predictive performance across multiple antimicrobial agents. Considering the results for the individual species, it is noticeable that *Acinetobacter baumannii* performs worst across the board. This can presumably be explained by the fact that this species has the lowest number of available samples, which may limit the model’s ability to learn stable patterns, and potentially by increased heterogeneity and complexity of resistance mechanisms observed in this organism.

### 4.4 Feature importance

The performance gain from the stacked model likely results from its ability to incorporate cross-resistance patterns. By using first-layer predictions from all antimicrobial agents as input to the second-layer model, the architecture allows the model to detect co-resistance patterns and shared genetic determinants. The feature importance analysis supports this interpretation: For the classes Carbapanems and Aminoglycosides, an increased feature importance within the classes was observed in the majority of cases (5 out of 6). Ertapenem and Meropenem belonging to the Carbapanems were also among the agents that benefited most from the stacked RF model compared to the one-layer approach. This suggests that the models have learned to exploit within-class resistance relationships in specific instances to improve the prediction. Incorporating these biological information via the second layer has likely contributed to the improved performance compared to the one-layer model. Similarly, previous work has shown that integrating structural information of antimicrobial agents can improve prediction accuracy, further highlighting the potential benefits of modeling relationships between drugs (Ryu *et al*. 2024). Considering the low feature importance within the Cephalosporins and Fluoroquinolones, these relationships could not yet be learned for these classes or are generally not as relevant as a feature for these predictions. Examining the third-generation Cephalosporins Cefotaxime and Ceftriaxone (Arumugham *et al*. 2023), there is no dependence in feature importance, despite their similar characteristics. This could be related to the fact that there are relatively few samples in the dataset that contain results for both agents. This suggests that more coherent data is needed in order to exploit correlations within these classes. Since the species was only used as a low-weight feature for 5 antimicrobial agents, the second layer hardly seems to put the learned antimicrobial agent information into a species-specific context. Given that the species information was already available to the first layer, it may also be due to the fact that it was already incorporated in the output of the first layer.

### 4.5 Validation dataset

The prediction of the validation dataset with the stacked model showed slightly decreasing median values compared to the cross-validation, with an accuracy of 0.938, ROC AUC of 0.971, and F1-score of 0.906. A stronger deviation of the weighted F1-score of 0.847 was observed, accompanied by a very wide confidence interval. This increased uncertainty is likely a result of the smaller sample size compared to the test results based on cross-validation which are stabilized by aggregation across multiple folds. While accuracy and ROC AUC are largely unaffected by small changes in sample size and class imbalance, indicated by their smaller CIs, the F1-score is highly sensitive to these variations.

It is noticeable that the values of a few antimicrobial agents have deteriorated significantly, although the vast majority still shows good results. Cefotaxime and Ertapenem are particularly remarkable in this regard. The drop of accuracy and F1-score for Cefotaxime while ROC AUC remained largely unaffected, suggests that these decreases reflect threshold-sensitive effects in minority-class prediction rather than a fundamental drop in discriminatory power. The results for Ertapenem additionally show a significantly worse ROC AUC value, which may be due to under-representation in the training data and shows that the model might overfit for individual antimicrobial agents. Although the F1-score indicates that the decision threshold was not always set optimally due to missing data, the ROC AUC, which is ≥ 0.90% in all but one case, suggests that the model still discriminates well. Furthermore, the accuracy, which is a ≥ 0.95% in 7 cases, demonstrates that the stacked model can provide very good performance for many antimicrobial agents. Further data with results for as many antimicrobial agents per sample as possible and balanced classes are needed in order to achieve these excellent results for all agents.

### 4.6 Comparison

Compared to previously published AMR prediction tools, the stacked RF model demonstrates a superior overall performance, despite being based only on relatively elementary information (i.e. the presence of AMR genes). In order to enable comparisons with as many other publications as possible, the frequently used median was used instead of the more informative weighted median. A recent review reported best-case results of up to 0.96 ROC AUC and 0.88 F1-score across high-quality studies (Abhadionmhen *et al*. 2025). Even the median results of the validation dataset prediction have a slightly higher ROC AUC and F1-score than these best cases. See Table 8 for several recent studies in comparison to stackPredAMR. This shows that Noman *et al*. (2023) even outperforms stackPredAMR with a median accuracy of 0.99, but makes only predictions for one species and the F1-score is lower. Pan *et al*. (2025) also has an accuracy of 0.99, but is limited to the closely related *E. cloacae* complex and 5 antimicrobial agents. All other evaluations in the comparison show lower scores. In contrast, our stacked model achieved a median ROC AUC of 0.97, a median accuracy of 0.94, and a median F1-score of 0.91, based on multi-class predictions (S/I/R) and multi-species data. These results suggest that the proposed approach not only matches but exceeds the performance of existing models, while also offering broader applicability to diverse species and resistance phenotypes. Comparing our results, such as Sundsfjord and Giske (2022), with the corresponding International Organization for Standardization (ISO) standard for most drug–bug combinations, which specifies an ME and VME of ≤3.00, 5 agents meet the standard, which further demonstrates the validity of the method.

**Table 8:** Performance comparison with results reported in previously published studies.^a^

Reference	Species	Accuracy	ROC AUC	F1 Score	#Antimicrobial agents
Noman *et al.* (2023)	*P. aeroginosa*	0.99		0.83	12
Pan *et al.* (2025)	*E. cloacae* complex	0.99			5
Kim *et al.* (2020)	*E. coli*, *K. pneumoniae*, *A. baumannii* + 6	0.91^b^			29
Jaillard *et al.* (2020)	*K. pneumoniae*		0.79		10
stackPredAMR	*E. coli*, *K. pneumoniae*, *A. baumannii*	0.94	0.97	0.91	18

a Values for competing methods are taken directly from the respective publications. The median values were calculated where possible to ensure good comparability.

b Mean instead median.

### 4.7 Locally sequenced and phenotyped samples

The results of the stackPredAMR predictions for 20 samples of species that were not part of the BV-BRC data set are mixed. Good performance was observed for Amikacin, Gentamicin, and Tobramycin with an accuracy of >0.9, indicating that generalization across species and sources was achieved. These agents also performed well on the validation dataset with metrics consistently ≥ 0.90 in all but one case, which indicates a good generalization of the model beyond already trained species for these agents. The prediction for Cefotaxime, Ciprofloxacin, and Imipenem was poor with an accuracy of <0.4, suggesting that the key features for this species-agent combination are not yet represented by the model or the training data. Their poor metrics in the validation data, where at least one was always <0.9, also indicate that the model provides insufficient results for these agents, regardless of species. With accuracies between 0.4 and 0.9, the remaining three antimicrobial agents also have potential for improvement. Overall, it becomes apparent that agents that already performed well in the validation data set also deliver good results in the locally sequenced sample, which may indicate good cross-species generalization of the model with sufficient training data. To draw generally valid conclusions about the performance, the number of samples would have to be significantly increased. Nevertheless, stackPredAMR can predict results for species not included during training.

### 4.8 Limitations and outlook

This study is limited to three bacterial species, 18 antimicrobial agents, and the use of AMR gene presence as the sole input feature, without incorporating information on regulatory activity. While this approach yields strong predictive performance, it may miss resistance mechanisms that depend on gene regulation rather than gene presence alone. That the ISO thresholds for most agents have not yet been met demonstrates that the model still provides insufficient results for clinical use. Additionally, although the model performs well on the current dataset, we only started to scratch the surface regarding its robustness on additional species and across broader resistance profiles. Furthermore, the model was evaluated using a random holdout strategy, and its generalizability across geographically distinct datasets or independent studies remains to be systematically assessed. The method was also developed using isolate genomes and has not yet been tested on metagenomic data, which could significantly reduce the time to result in clinical workflows but introduces new challenges. These limitations open clear avenues for future work: expanding the model to a broader range of pathogens and drugs, adapting it to metagenomic applications, and integrating gene regulatory data.

## 5 Conclusion

stackPredAMR achieves a median accuracy of 0.94, ROC AUC of 0.97, and F1-score of 0.91 measured against real-world validation data, in some cases outperforming previously published methods. These results suggest that the stacked approach leveraging cross-resistance patterns enhances AMR prediction. The resistance patterns for all antimicrobial agents could be predicted with ≥ 0.90% ROC AUC in all but one case. An accuracy of ≥ 0.95% was achieved for 7 agents. These above-average results were achieved solely through training on AMR gene presence. The regulatory information of the original sequence was disregarded entirely. However, we see great potential in incorporating this additional information in the future. Another unique feature of this work is the high degree of flexibility of our pipeline. Additional antimicrobial agents and species can easily be integrated as part of future endeavors. A potential long-term application of such a prediction pipeline would be one that eliminates the need for time-consuming species isolation and enables direct prediction based on metagenomic data. This could greatly reduce the workload in everyday clinical practice and speed up the detection of resistance. Future work should explore the integration of gene regulatory information and expand to a broader range of species and antimicrobial agents. It should also adapt the approach to metagenomic data, where prediction from mixed samples could reduce diagnostic turnaround times and improve applicability in clinical and surveillance settings.

## Supplementary Material

vbag153_Supplementary_Data

## Data Availability

Source code for preprocessing and model training is available on GitHub (https://github.com/IKIM-Essen/WIN-KID/tree/v1.0.0.0) under a BSD-style license. The list of BV-BRC sample IDs used (samples_used.csv) and the list of input features (feature_list.csv) can be downloaded from GitHub. Source code and results of the CARD-driven reference approach is available on GitHub (https://github.com/IKIM-Essen/WIN-KID/releases/download/v1.0.0.0/CARD_Reference.zip).
