# Supplementary material for: stackPredAMR—a stacked random forest approach improves AMR phenotype prediction for multiple species and antimicrobial agents
Source: Bioinform Adv. 2026 Jun 2;6(1):vbag153. doi: 10.1093/bioadv/vbag153 (PMC13342711; doi:10.1093/bioadv/vbag153)
Supplement: vbag153_Supplementary_Data [file vbag153_supplementary_data.zip › stackPredAMR_28052026_sup_V2.pdf]

Supplemental Information

1 CARD-driven reference results

	Accuracy	ROC AUC	F1 Score
**Amikacin**	0.157	0.258	0.501
**Ampicillin**	0.933	0.966	0.515
**Aztreonam**	0.311	0.005	0.501
**Cefazolin**	0.852	0.889	0.861
**Cefepime**	0.681	0.809	0.508
**Cefotaxime**	0.794	0.799	0.818
**Ceftazidime**	0.370	0.538	0.502
**Ceftriaxone**	0.810	0.894	0.517
**Cefuroxime**	0.257	0.076	0.501
**Ciprofloxacin**	0.560	0.710	0.453
**Ertapenem**	0.588	0.740	0.500
**Gentamicin**	0.360	0.512	0.497
**Imipenem**	0.707	0.827	0.507
**Levofloxacin**	0.476	0.586	0.454
**Meropenem**	0.756	0.144	0.537
**Norfloxacin**	0.156	0.260	0.505
**Tobramycin**	0.565	0.681	0.581
**Trimethoprim**	0.670	0.801	0.486
**Median**	0.577	0.696	0.503
**WT Median**	0.560	0.586	0.502

Supplementary Table 1: Evaluation metrics of the CARD-driven reference classification across all antimicrobial agents, with the overall median displayed before the last row. The last row shows the weighted median, which was weighted based on the total number of test samples for each individual agent. Cells are tint-coded according to performance thresholds: dark (>0.95), light (>0.85-0.95) and white ($\leq$ 0.85).

2 Runtime

The execution time using 5-fold cross-validation was measured for both models. The one-layer RF model completed in 32.4 seconds total (30.4s user 0.7s system), while the stacked RF model required 329.7 seconds (326.9s user 1.7s system). The training of the stacked model for the validation dataset completed in 86.6 seconds (83.8 user 1.0s system) and the prediction required 11.9 seconds (10.2s user 0.6s system). All experiments were conducted on a MacBook Air equipped with an Apple M3 chip and 24 GB RAM, and where additionally executed on a Linux system. The reported times reflect the execution duration for preprocessing, model training, testing and evaluation, excluding the interpretation of MIC values and annotation of AMR genes.

3 Hyperparameter Tuning

3.1 Procedure

The hyperparameter tuning was performed using CV for all antimicrobial agents collectively on the training dataset. Initially the one-layer RF was tested with all combinations of the parameters listed below (See 3.2). The combination with the best metrics was then used for further experiments and is listed under 3.3. The second layer of the stacked RF was tested in the same way, retaining the optimal values for the first layer from the one-layer RF. In addition, the following splitting types were tested for the internal OOF validation: random, stratify and cluster. Stratified splitting and the parameters under 3.4 delivered the best results and were used for further analysis.

3.2 List of tested parameters

- `n_estimators: 10, 100, 200, 1000, 1500`
- `class_weight: "balanced", "balanced_subsample"`
- `max_depth: 10, 20, 50`
- `min_samples_split: 2, 5`
- `min_samples_leaf: 1, 2, 4`
- `max_features: "log2", 0.3`
- `bootstrap: True, False`

3.3 RF parameters first layer

- `n_estimators = 100`
- `class_weight = "balanced_subsample"`
- `max_depth = 20`
- `min_samples_split = 2`
- `min_samples_leaf = 2`

- max_features = 0.3
- bootstrap = True

3.4 RF parameters second layer

- n_estimators = 1500
- class_weight = "balanced_subsample"
- max_depth = 20
- min_samples_split = 2
- min_samples_leaf = 2
- max_features = 0.3
- bootstrap = True

4 EUCAST values

	Enterobacterales		Acinetobacter	
	S $\leq$	R $>$	S $\leq$	R $>$
**Amikacin**	8	8	8	8
**Ampicillin**	8	8	-	-
**Aztreonam**	1	4	-	-
**Cefazolin**	0.001	4	-	-
**Cefepime**	1	4	-	-
**Cefotaxime**	1	2	-	-
**Ceftazidime**	1	4	-	-
**Ceftriaxone**	1	2	-	-
**Cefuroxime**	0.001	8	-	-
**Ciprofloxacin**	0.25	0.5	0.001	1
**Ertapenem**	0.5	0.5	-	-
**Gentamicin**	2	2	4	4
**Imipenem**	2	4	2	4
**Levofloxacin**	0.5	1	0.5	2
**Meropenem**	2	8	2	8
**Norfloxacin**	0.5	0.5	-	-
**Tobramycin**	2	2	4	4
**Trimethoprim**	4	4	-	-

Supplementary Table 2: EUCAST v15 clinical breakpoints in mg/L used to determine the resistance class from MIC values.

5 Sample weighted median CI

	Accuracy	ROC AUC	F1 Score
**WT. Median One Layer RF CV**	0.939 – 0.959	0.965 – 0.983	0.832 – 0.939
**WT. Median Stacked RF CV**	0.946 – 0.960	0.969 – 0.985	0.847 – 0.934
**WT. Median Stacked RF Validation**	0.904 – 0.946	0.967 – 0.971	0.659 – 0.939

Supplementary Table 3: 95% confidence intervals of the sample weighted medians for the one layer RF CV, stacked RF CV and stacked RF validation across the evaluation metrics

6 Feature importance

	CAZ	CFZ	CRO	CTX	CXM	FEP	ETP	IPM	MEM	CIP	LVX	NOR	AMI	GEN	TOB	AMP	AZT	TMP	Species
**CAZ**	0.64	0.01	0.01	0.08	0.01	0.02	0.02	0.00	0.01	0.06	0.02	0.00	0.03	0.02	0.02	0.01	0.04	0.01	0.00
**CFZ**	0.09	0.52	0.02	0.05	0.12	0.01	0.01	0.01	0.01	0.01	0.01	0.00	0.01	0.02	0.02	0.00	0.08	0.00	0.00
**CRO**	0.02	0.01	0.51	0.00	0.00	0.05	0.03	0.00	0.01	0.03	0.03	0.16	0.00	0.00	0.00	0.00	0.01	0.13	0.00
**CTX**	0.14	0.00	0.00	0.63	0.07	0.04	0.01	0.00	0.01	0.02	0.01	0.00	0.00	0.00	0.02	0.00	0.04	0.00	0.00
**CXM**	0.09	0.14	0.02	0.02	0.47	0.01	0.01	0.01	0.01	0.01	0.01	0.00	0.01	0.01	0.03	0.00	0.15	0.00	0.00
**FEP**	0.04	0.02	0.10	0.03	0.03	0.39	0.03	0.01	0.02	0.05	0.04	0.05	0.02	0.02	0.02	0.01	0.07	0.04	0.00
**ETP**	0.03	0.01	0.02	0.02	0.02	0.02	0.18	0.47	0.11	0.02	0.02	0.00	0.02	0.01	0.01	0.02	0.02	0.00	0.00
**IPM**	0.01	0.01	0.00	0.01	0.02	0.01	0.04	0.57	0.15	0.03	0.03	0.00	0.03	0.02	0.04	0.01	0.01	0.00	0.01
**MEM**	0.03	0.01	0.01	0.01	0.01	0.02	0.05	0.15	0.41	0.03	0.04	0.00	0.07	0.04	0.03	0.00	0.02	0.00	0.07
**CIP**	0.11	0.01	0.01	0.03	0.00	0.02	0.02	0.02	0.03	0.59	0.04	0.00	0.04	0.02	0.02	0.01	0.01	0.00	0.01
**LVX**	0.03	0.01	0.02	0.01	0.04	0.04	0.03	0.02	0.04	0.13	0.53	0.00	0.02	0.02	0.01	0.01	0.03	0.00	0.00
**NOR**	0.08	0.00	0.06	0.00	0.00	0.13	0.00	0.00	0.02	0.28	0.00	0.36	0.01	0.01	0.01	0.02	0.00	0.02	0.00
**AMI**	0.14	0.00	0.00	0.01	0.00	0.02	0.01	0.01	0.04	0.02	0.01	0.00	0.56	0.11	0.02	0.00	0.01	0.00	0.01
**GEN**	0.09	0.01	0.00	0.01	0.00	0.01	0.01	0.01	0.02	0.02	0.01	0.00	0.07	0.68	0.05	0.00	0.01	0.00	0.00
**TOB**	0.05	0.01	0.00	0.01	0.01	0.01	0.01	0.02	0.03	0.03	0.01	0.00	0.05	0.12	0.62	0.00	0.03	0.00	0.00
**AMP**	0.13	0.01	0.04	0.01	0.01	0.03	0.04	0.01	0.01	0.03	0.02	0.04	0.01	0.01	0.09	0.48	0.01	0.03	0.00
**AZT**	0.12	0.09	0.01	0.01	0.09	0.03	0.01	0.01	0.02	0.03	0.02	0.00	0.02	0.01	0.02	0.00	0.50	0.01	0.00
**TMP**	0.04	0.00	0.05	0.01	0.01	0.05	0.02	0.00	0.03	0.05	0.00	0.03	0.02	0.02	0.02	0.09	0.04	0.52	0.00

Supplementary Table 4: Mean feature importance of the second layer CV of the stacked RF model. Rows list the antimicrobial agents for which the models were trained. Columns represent the features, consisting of all antimicrobial agents and the bacterial species information. Each cell indicates the contribution of the corresponding feature to the prediction of the given antimicrobial agent model.

7 Evaluation metrics per species

Accuracy			
**Target**	***E. coli***	***K. pneumoniae***	***A. baumannii***
**Amikacin**	0.977	0.929	0.818
**Ampicillin**	0.932	0.994	
**Aztreonam**	0.838	0.956	
**Cefazolin**	0.938	0.976	
**Cefepime**	0.838	0.903	
**Cefotaxime**	0.989	0.985	
**Ceftazidime**	0.959	0.952	
**Ceftriaxone**	0.961	0.993	
**Cefuroxime**		0.979	
**Ciprofloxacin**	0.969	0.934	0.935
**Ertapenem**	0.883	0.942	
**Gentamicin**	0.990	0.928	0.924
**Imipenem**		0.951	0.733
**Levofloxacin**	0.891	0.921	0.959
**Meropenem**	0.996	0.899	0.870
**Norfloxacin**	0.953		
**Tobramycin**	0.995	0.938	0.870
**Trimethoprim**	0.897	0.926	
**Median**	0.956	0.942	0.870

Supplementary Table 5: Accuracy per species of the stacked RF CV classification across all antimicrobial agents, with the overall median shown in the last row. Cells are tint-coded according to performance thresholds: dark (>0.95), light (>0.85-0.95) and white ($\leq$ 0.85).

ROC AUC			
**Target**	***E. coli***	***K. pneumoniae***	***A. baumannii***
**Amikacin**	0.989	0.973	0.863
**Ampicillin**	0.956	0.929	
**Aztreonam**	1.000	0.970	
**Cefazolin**	0.700	0.989	
**Cefepime**	0.943	0.959	
**Cefotaxime**	0.997	0.988	
**Ceftazidime**	0.989	0.974	
**Ceftriaxone**	0.986	0.985	
**Cefuroxime**		0.984	
**Ciprofloxacin**	0.966	0.959	0.908
**Ertapenem**	0.786	0.982	
**Gentamicin**	0.993	0.968	0.859
**Imipenem**		0.981	0.906
**Levofloxacin**	0.942	0.980	0.849
**Meropenem**	0.989	0.952	0.821
**Norfloxacin**	0.960		
**Tobramycin**	0.994	0.976	0.900
**Trimethoprim**	0.921	0.986	
**Median**	0.976	0.976	0.863

Supplementary Table 6: ROC AUC per species of the stacked RF CV classification across all antimicrobial agents, with the overall median shown in the last row. Cells are tint-coded according to performance thresholds: dark (>0.95), light (>0.85-0.95) and white ($\leq$ 0.85).

F1 Score			
**Target**	***E. coli***	***K. pneumoniae***	***A. baumannii***
**Amikacin**	0.817	0.908	0.705
**Ampicillin**	0.882	0.878	
**Aztreonam**	0.581	0.937	
**Cefazolin**	0.484	0.972	
**Cefepime**	0.772	0.687	
**Cefotaxime**	0.987	0.899	
**Ceftazidime**	0.923	0.878	
**Ceftriaxone**	0.960	0.848	
**Cefuroxime**		0.970	
**Ciprofloxacin**	0.801	0.649	0.876
**Ertapenem**	0.468	0.933	
**Gentamicin**	0.981	0.927	0.867
**Imipenem**		0.856	0.652
**Levofloxacin**	0.757	0.877	0.689
**Meropenem**	0.599	0.746	0.593
**Norfloxacin**	0.936		
**Tobramycin**	0.993	0.935	0.866
**Trimethoprim**	0.887	0.900	
**Median**	0.849	0.899	0.705

Supplementary Table 7: F1 Score per species of the stacked RF CV classification across all antimicrobial agents, with the overall median shown in the last row. Cells are tint-coded according to performance thresholds: dark (>0.95), light (>0.85-0.95) and white (≤ 0.85).

8 VME and ME of stacked RF validation

	VME	ME
**Amikacin**	4.41	1.44
**Ampicillin**	0.17	4.35
**Aztreonam**	0.51	3.18
**Cefazolin**	0.15	2.62
**Cefepime**	1.59	7.85
**Cefotaxime**	0.00	41.48
**Ceftazidime**	0.86	2.10
**Ceftriaxone**	2.37	0.00
**Cefuroxime**	0.00	1.37
**Ciprofloxacin**	0.91	5.82
**Ertapenem**	1.07	25.17
**Gentamicin**	2.31	3.09
**Imipenem**	1.35	10.56
**Levofloxacin**	1.84	4.76
**Meropenem**	1.31	7.21
**Norfloxacin**	0.00	2.70
**Tobramycin**	4.27	2.29
**Trimethoprim**	6.96	5.22
**Median**	1.19	3.77

Supplementary Table 8: VME and ME of the stacked RF validation classification across all antimicrobial agents for the validation data set. Cells are tinted if the corresponding values are ≤ 3.00
